# A patient-level data meta-analysis of standard-of-care treatments from eight prostate cancer clinical trials

**DOI:** 10.1038/sdata.2016.27

**Published:** 2016-05-10

**Authors:** N. Geifman, A.J Butte

**Affiliations:** 1 Institute for Computational Health Sciences, University of California San Francisco, Mission Hall, 550 16th Street, San Francisco, CA 94158-2549, USA

**Keywords:** Health care, Metabolomics, Metabolomics

## Abstract

Open clinical trial data offer many opportunities for the scientific community to independently verify published results, evaluate new hypotheses and conduct meta-analyses. These data provide valuable opportunities for scientific advances in medical research. Herein we present the comparative meta-analysis of different standard of care treatments from newly available comparator arm data from several prostate cancer clinical trials. Comparison of survival rates following treatment with mitoxantrone or docetaxel in combination with prednisone as well as prednisone alone, validated the previously demonstrated superiority of treatment with docetaxel. Additionally, comparison of four testosterone suppression treatments in hormone-refractory prostate cancer revealed that subjects who had undergone surgical castration had significantly lower survival rates than those treated with LHRH, anti-androgen or LHRH plus anti-androgen, suggesting that this treatment option is less optimal. This study illustrates how the use of patient-level clinical trial data enables meta-analyses that can provide new insights into clinical outcomes of standard of care treatments and thus, once validated, has the potential to help optimize healthcare delivery.

## Introduction

Prostate cancer is one of the most common malignancies in men and a leading cause of illness and death in industrialized countries^1^. This type of malignancy is heterogeneous in nature and consists of both hormone-responsive and hormone-independent cells. Earlier diagnosis has meant that many patients are treated while the cancer is still in its localized form. This localized form of prostate cancer may be managed by a variety of different approaches including prostatectomy, various forms of radiotherapy and watchful waiting. A small percentage of subjects, however, are diagnosed or eventually develop an advanced form of the disease; and even though radical prostatectomy and radiation therapy can help control the disease, in over a third of patients elevated prostate-specific antigen (PSA) levels will recur^2,3^. These patients are often treated with hormonal therapy such as Luteinizing Hormone Release Hormone (LHRH) agonists or anti-androgens which reduce the level of serum testosterone. Following therapy of curative intent, androgen ablation by hormonal treatment or by surgical castration (orchiectomy) in subjects with hormonally sensitive prostate cancer may lead to significant reduction in the risk of disease recurrence and increase overall survival^4,5^. Moreover, in the majority of patients with metastatic disease, androgen ablation has been shown to improve symptoms and lead to regression of metastases and a lowering of serum PSA levels^6–8^. While the benefits of hormonal or surgical castration are well documented, to the best of our knowledge, the different approaches have not been systematically compared to each other.

All patients with metastatic disease become un-responsive to hormonal therapy with time. Tumors treated with anti-androgen therapy eventually become androgen-independent and start to grow again^6,9^. In cases where the disease continues to progress even though testosterone ablation by surgical or hormonal treatment has been achieved, the disease is referred to as Hormone-Refractory Prostate Cancer (HRPC).

Since metastatic HRPC is incurable, treatments for this form of the disease focus on relieving symptoms, prolonging and improving the quality of the patient’s life. Currently, the approved chemotherapy treatment for HRPC patients is a combination of prednisone with mitoxantrone or docetaxel. While survival differences between different chemotherapy treatments in HRPC have been previously demonstrated, this work aims to validate those findings while taking a more systematic approach for comparison.

Many cancer clinical trials are conducted each year; recently, the scientific community’s demand for open sharing of data from these trials has encouraged sharing of patient-level data from clinical trials^10–12^. One such clinical trial data sharing platforms is the Project Data Sphere^10,11^ which aims at making raw data from cancer clinical trials available for research. The database currently holds data from the comparator arms of 55 cancer clinical trials. In its current form, data pertaining only to subjects assigned to the comparator arms of cancer clinical trials (usually receiving standard of care treatment) are made available. Nevertheless, this data resource has the potential to be very useful to the community.

Herein we present the meta-analysis and comparison of prostate cancer standard-of-care treatments from the control, or comparator, arms of eight clinical trials including 4127 patients. By combining patient-level data from comparator arms across different trials we were able to validate previously published results as well as identify adverse survival outcomes for surgical castration therapy in prostate cancer. While these findings are for hypothesis-generation only, this work aims to demonstrate how meta-analysis of comparator arms from clinical trials can provide new insights into clinical outcomes.

## Results

### A meta-dataset of clinical trial comparator arms

**Figure 1:**
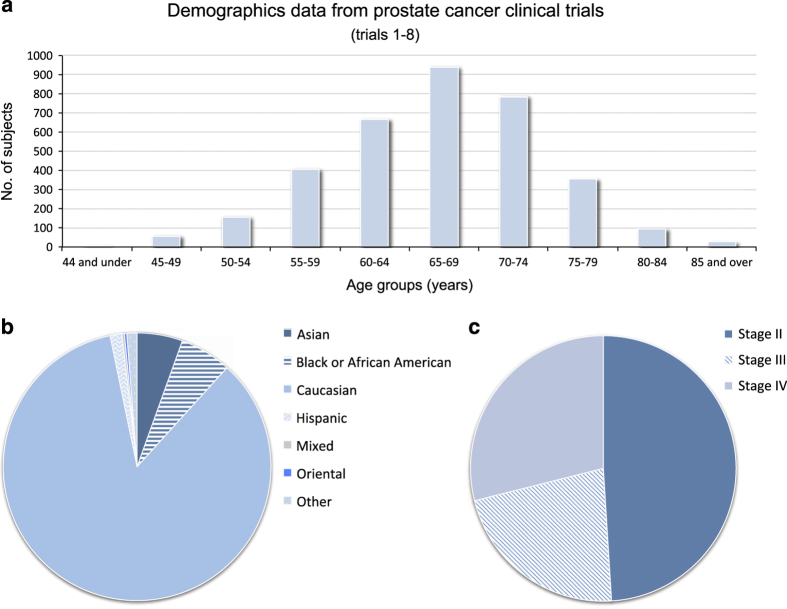
Demographics data from eight prostate cancer clinical trials (where data were available). (**a**) Age group distribution. (**b**) Racial group distribution, using the original terms from these studies. (**c**) Cancer stage distribution.

Patient-level data from eight prostate cancer clinical trials, with a total of 4127 subjects, were combined, integrated and used for this study. The mean age of subjects included in these trials was 67.3 years (Fig. 1a), the majority (85%) were Caucasian (Fig. 1b), and most were diagnosed with stage II or stage III cancer (Fig. 1c). Overall, 868 (21%) subjects were reported to have died during the studies and follow-up.

### A comparison of chemotherapies combined with prednisone and treatment with prednisone alone

**Figure 2:**
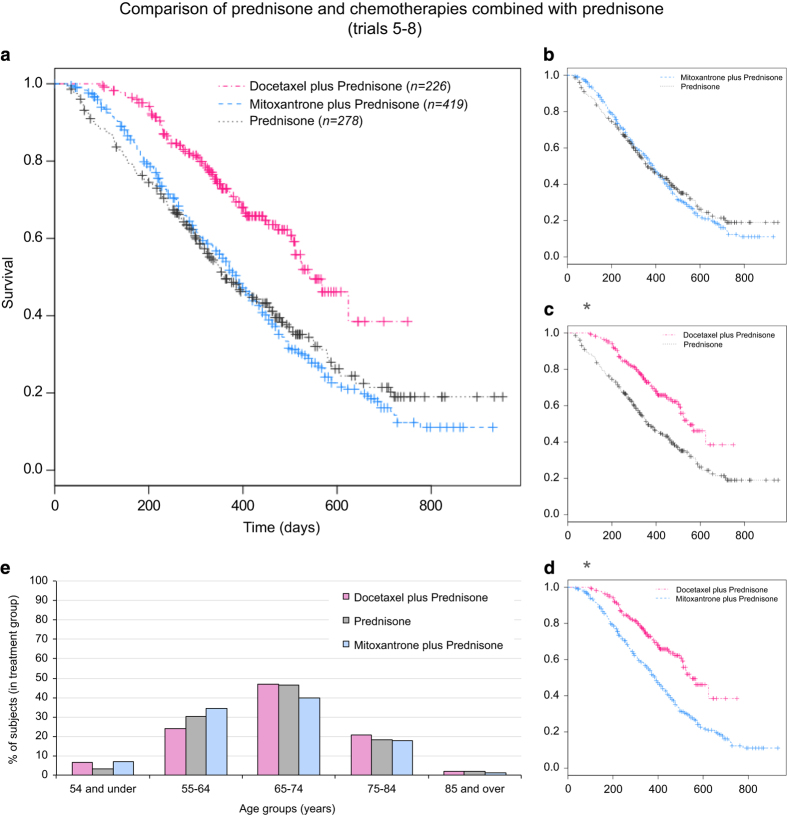
Comparison of prednisone and chemotherapies combined with prednisone. (**a**–**d**) Survival of prostate cancer subjects in three different treatment groups (docetaxel plus prednisone: *n*=226, mitoxantrone plus prednisone: *n*=419 and prednisone: *n*=278). * Indicates groups that are significantly different (Cox proportional regression). (**e**) Age group distribution in the three treatment groups.

We compared the survival of a subset of subjects from three comparator treatment groups (a total of 923 subjects), two combining different chemotherapies with prednisone and one of prednisone alone, while controlling for age, race and the study from which the data originated (Fig. 2a). Similar age group distribution was found between the three treatment groups (Fig. 2e), all subjects are male and all have stage IV prostate cancer. No survival differences were found between racial groups within each treatment group (Supplementary Fig. 1). Comparison of the survival of subjects from different clinical trials but receiving the same treatment (mitoxantrone plus prednisone, the only treatment group combining data from 2 different trials) showed no significant differences between the trials (Supplementary Fig. 2) suggesting it was safe to combine these data. Our results reveal that there are no significant differences in survival of subjects treated with mitoxantrone plus prednisone to those treated with prednisone alone (with estimated survival probabilities of 0.54 and 0.495, respectively, at 1 year follow-up and 0.123 and 0.19, respectively, at 2 years follow-up, Fig. 2b). On the other hand, subjects treated with a combination of docetaxel plus prednisone showed significantly higher survival (*P*<0.001) than those treated with prednisone alone (0.73 in docetaxel plus prednisone treated subjects at 1 year follow-up and 0.385 at 2 years follow-up, Fig. 2c). Survival rates are also significantly (*P*<0.001) higher in the docetaxel plus prednisone treated group in comparison to mitoxantrone plus prednisone treated group (Fig. 2d).

### Surgical castration shows lower survival rates than hormonal therapy

**Figure 3:**
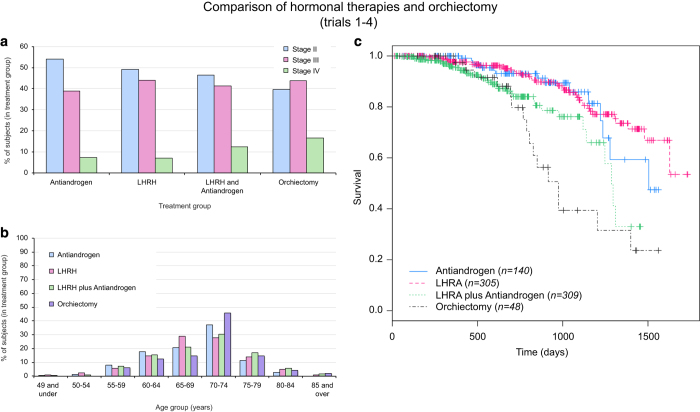
Comparison of hormonal therapies and orchiectomy. (**a**) Cancer stage distribution in the four treatment groups (where data were available). (**b**) Age group distribution in the four treatment groups (where data were available). (**c**) Survival of prostate cancer subjects in four different treatment groups (antiandrogen: *n*=140, LHRH: *n*=305, LHRA plus antiandrogen: *n*=309 and orchiectomy: *n*=48).

We next compared the survival of three hormonal testosterone suppression treatment groups and surgical castration (orchiectomy), totaling with 802 subjects. The majority of subjects in all treatment groups were diagnosed with stage II or stage III (Fig. 3a). Some differences in survival were seen between the original clinical trials within the LHRH and the anti-androgen treatment groups, however these were not statistically significant when controlling for age, race and cancer stage (Supplementary Fig. 3). Nevertheless, these differences were accounted for in our Cox proportional regression model used for calculating survival differences between the treatment groups. Similar age group distributions were found between treatment groups (Fig. 3b) and the vast majority of the subjects (90%) were Caucasian. Our results reveal that there are no differences in survival between subjects treated with anti-androgen or LHRH (with estimated survival probabilities of 0.858 and 0.837, respectively, at 3 years follow-up and 0.593 and 0.713, respectively, at 4 years follow-up, Fig. 3c). On the other hand, the survival of subjects treated with surgical castration was significantly lower (0.394 at 3 years follow-up and only 0.236 at 4 years follow-up) than those in any of the other treatment groups (*P<*0.05) even when controlling for age, cancer stage, race and study. Additionally, the survival of subjects treated LHRH plus anti-androgen was nominally lower (0.761 at 3 years follow-up) than those of subjects treated with LHRH or anti-androgen alone. Similar adverse events were reported for each of the treatment groups; however, proportions for these adverse events varied (Table 1).

## Discussion

The standard of care implemented for a given cancer type can vary dramatically amongst providers. The availability of data from multiple clinical trials offers the unprecedented opportunity to compare outcomes from different standard of care treatments. To evaluate differences in standard-of-care treatment for prostate cancer, we present here the analysis of comparator arm patient-level data from a newly available open clinical trial dataset. Using this dataset, known outcome differences for different chemotherapy treatments for hormone-refractory prostate cancer are further validated. By contrast, there is no general agreement on the best method for reducing testosterone levels in prostate cancer patients, and we were able here to gain insight into comparative survival outcomes of four different testosterone suppression treatment options.

In our first analyses we compare the survival outcomes between two chemotherapies in combination with prednisone as well as prednisone alone showing a better outcome after treatment with the docetaxel plus prednisone combination. Our findings are in line with the results of a separate phase 3 clinical trial, where treatment with docetaxel plus prednisone in the first-line treatment of patients with castration resistant prostate cancer was shown to have a 2.4 month survival benefit compared to mitoxantrone plus prednisone, prolonging survival from 16.5 months to 18.9 months^13^.

As a result of this trial, docetaxel every 3 weeks in combination with daily prednisone was approved for the treatment of patients with hormone-refractory metastatic prostate cancer, and is now the accepted standard of care for these patients^14^. The meta-analysis conducted here further demonstrates the survival benefits of treatment with docetaxel plus prednisone in comparison to mitoxantrone plus prednisone or prednisone alone. However, since subjects in the mitoxantrone plus prednisone and the prednisone treatment groups were selected after failing treatment with docetaxel, it is possible that they are generally less likely to respond to treatment and this contributed to the lower survival seen in these groups. We additionally show no survival advantages for treatment with mitoxantrone plus prednisone in comparison to prednisone alone, similarly to previous studies^15,16^. While palliative effects were also observed in HRPC patients following the administration of either corticosteroid alone^17^ or, mitoxantrone with either prednisone or hydrocortisone^16,18–20^, these were even more significant in subjects treated with docetaxel^13^. In addition to validating other trial results, this analysis demonstrates the power that meta-analyses have in detecting therapeutic advantages.

One of the goals in treating prostate cancer is achieving and maintaining an effective suppression of testosterone levels. This can be achieved by administration of LHRH agonists, anti-androgens or by surgical castration. While with bilateral orchiectomy, lowered testosterone levels are almost instantly achieved, it is not commonly performed mostly due the irreversibility of the procedure^21^. In an early prostate cancer study, treatment with orchiectomy, placebo, estrogen or orchiectomy plus estrogen was compared. In stage IV patients, equivalent survival rates at nine years follow-up were seen in all four arms suggesting no survival differences between treatments in this subset of patients^22,23^. In a second trial, orchiectomy did not differ from androgen-suppression therapy in survival and response to chemotherapy in patients with hormone-refractory prostate cancer^24^. Our results however, indicate that orchiectomy is less beneficial due to lower survival rates in comparison to modern hormonal therapies (i.e., other than estrogen) even when controlling for age, cancer stage, race and the study from which the data originated. While there are better quality-of-life outcomes such as less frequently reported erectile dysfunction, breast swelling and physical discomfort for orchiectomy in comparison to patients treated with LHRH^25,26^, the lower survival rates demonstrated here should be considered in the decision to undergo surgical castration following further validation of our results. Additionally, our results indicate the trend that subjects treated with LHRH and anti-androgen have lower survival rates than those treated with either LHRH or anti-androgen alone. Anti-androgens may be added to treatment when the LHRH agonist (or other androgen lowering treatment) is no longer working well on its own or it may be given along with an LHRH agonist as initial therapy (referred to as combined androgen blockade). Meta-analysis of the literature showed that combined androgen blockade had no survival benefits in comparison to treatment with LHRH agonists or orchiectomy at 2 years, and better survival at 5 years^27^. This literature meta-analyses compared combined androgen blockade therapy to all monotherapies (LHRH agonists or orchiectomy) grouped as one, which could explain the difference from our results. Additionally, none of the trials included in these previous meta-analyses included subjects treated with bicalutamide as the anti-androgen while in our analysis at least 115 of the 309 subjects in the LHRH plus anti-androgen group were treated with bicalutamide plus some LHRH agonist. A clinical trial, specifically designed to compare the four androgen ablation treatment options should be considered in order to validate our own findings and to clarify which treatment approach is most favorable.

There are several limitations to the work presented here. First, only outcomes as measured by survival rates and adverse events are presented. Ideally, testosterone levels in each of the testosterone suppression treatment groups should have been examined and compared to clinically relevant levels agreed upon in the literature^21^, however these data were not fully available in this dataset. Second, some of the data lacks critical detail; for example, for many of the subjects we do not know which specific drugs (or dose) were given in each of the testosterone suppression treatments (such as goserelin or leuprorelin) since the data files only included general terms to describe each treatment arm (such as LHRH agonists). Third, while age, race and cancer stage were controlled for in the analyses presented here, there could be some other confounders not taken into account. Finally, significant effort had to be invested in organizing, annotating and integrating the data from the different trials before a comparative analysis could be performed. Since considerable manual effort needed to be invested in the processing of these data, only eight prostate cancer trials were downloaded and used as proof of concept for the work presented here. Additionally, only the control, placebo or comparator arms data were made available for research, making reanalysis of the original trials or a more comprehensive meta-analysis impossible. Nevertheless, the clinical trials dataset we describe here holds a lot of potential for cancer research and future work on this project will include adding data from all the trials available on the Project Data Sphere site and from other resources as they become available.

There are many potential benefits for the sharing of clinical trial data^28–30^; with trial data available to the scientific community, trial results can be examined, reproduced and combined with other data to conduct meta-analyses^31^. Comparing treatments, outcomes and other disease-related patterns by meta-analysis can help gain a better understanding of the disease under investigation. Furthermore, given a large enough number of studies pertaining to the same disease, the outcomes of different treatments may be compared. Additionally, these data may be used to identify and define subgroups of subjects who respond better to a specific treatment. The abundance of raw, patient-level clinical data has great potential in leading to scientific advances in medical research and development of new techniques in clinical informatics^32–34^. Availability of patient-level data such as raw clinical measurements, rather than summarized table data, can allow researchers to independently verify published results as well as evaluate new hypotheses.

To conclude, we present here the meta-analyses of data from newly available pharma-released clinical trials and the comparison of several lines of standard-of-care treatment in prostate cancer. By combining comparator arms from different trials we were able to validate previously described results as well as identify possible adverse survival outcomes for surgical castration therapy in prostate cancer. While potential refinements in standard of care are highly desirable outcomes of future meta-analyses of other clinical trial control data sets, we envision that availability of data from experimental arms from multiple clinical trials would enable meta-analyses that could lead to the discovery of experimental drug effects not observable in individual clinical trials.

## Methods

### Clinical trial data from the project data sphere portal

**Figure 4:**
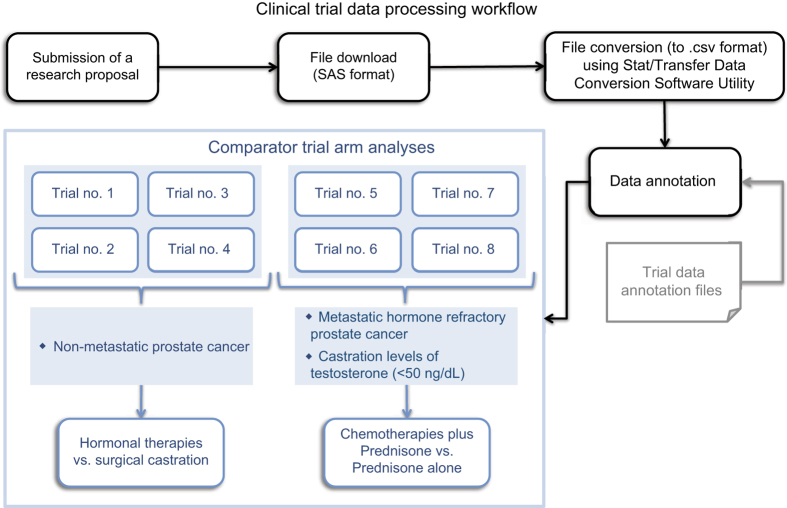
Clinical trial data processing workflow. The trial numbers refer to the numbers provided in Table 1. Inclusion criteria for trials 5,7 and 8 included failed past treatment with Docetaxel.

Clinical trial data were obtained from the Project Data Sphere portal (projectdatasphere.org) which stores, shares and allows analysis of patient level phase III cancer trial data^11^. Following registration, submission and approval of a research proposal, the data are made available for either download or analysis on an online SAS platform (Fig. 4).

Survival, demographic and treatment data for 8 prostate cancer clinical trials (of the first trials made available on the site that included annotation files, Data Citations 1-8, Table 2) were downloaded from the Project Data Sphere database and used for the analyses presented here, totaling 4127 comparator arm participants represented by patient-level data. Subsets of subjects with matching treatments were generated for the comparison analyses described herein. For the comparison of chemotherapies combined with prednisone and treatment with prednisone alone, a total of 923 subjects, for which treatment and survival data were available, were used. For the comparison of androgen ablation treatments a total of 802 subjects, for which treatment and survival data were available, were used.

### Assigning cancer stage to subjects with missing assessments

For several of the trials in our dataset (trials 1–4, Table 2), the cancer stage was not readily available for the subjects included in our analysis (a total of 802 subjects). We therefore assigned each subject with a cancer stage based on the available tumor status and involvement of lymph nodes and based on the AJCC cancer staging manual^35^. If there was any indication of metastasis in lymph nodes, stage IV was assigned to those subjects (regardless of tumor status). For subjects with no lymph node involvement and who had a tumor status of T1b, T1c, T1 or T2, stage II was assigned. For subjects with no lymph node involvement and a tumor status of T3, stage III was assigned. For subjects with no lymph node involvement and tumor status T4, stage IV was assigned. There were no other combinations of tumor and metastasis status in the data. A cancer stage was successfully assigned for 640 subjects.

### Statistical analyses

Kaplan-Meier analyses were conducted using the ‘Survival’ package (version 2.38–3) in R^36^. Cox proportional regression was used to calculate the significance for the differences between sample sets. These models controlled for differences in age, race, cancer stage and the clinical trial from which the data originated (where applicable). Chi-squared test was used for calculating differences between age group distributions.

### Code availability

The scripts used for all data analyses, implemented in R, are available in the Supplementary Code section of the Supplementary Information.

## Additional Information

**Disclaimer:** This publication is based on research using information obtained from www.projectdatasphere.org, which is maintained by Project Data Sphere, LLC. Neither Project Data Sphere, LLC nor the owner(s) of any information from the web site have contributed to, approved or are in any way responsible for the contents of this publication.

**How to cite this article:** Geifman, N. & Butte, A. J. A patient-level data meta-analysis of standard-of-care treatments from eight prostate cancer clinical trials. *Sci. Data* 3:160027 doi: 10.1038/sdata.2016.27 (2016).

## Supplementary Material

Supplementary Information

## Figures and Tables

**Table 1:** Adverse events in each of the hormonal therapies and orchiectomy groups.

**Orchiectomy (%)**	**Antiandrogen (%)**	**LHRH (%)**	**LHRH plus Antiandrogen (%)**
Vasodilation (8.3)	Male breast pain (7.1)	Gynecomastia (6.9)	Headache (5.5)
Gynecomastia (8.3)	Impotence (3.6)	Vasodilation (4.9)	Gynecomastia (4.5)
Nausea (6.3)	Rash (2.9)	Male breast pain (4.3)	Asthenia (4.2)
Rash (4.2)		Breast tenderness (3.6)	Vasodilation (3.9)
Face edema (4.2)		Hot flashes (3.3)	Rash (3.2)
Nipple tenderness (4.2)		Nausea (3)	Peripheral oedema (2.9)
Breast tenderness (4.2)		Impotence (3)	Diarrhea (2.6)
		Diarrhea (2.6)	Constipation (2.6)
			Rhinitis (2.6)
Adverse events (COSTART coding symbol terms and MedDRA terms) reported as caused by the treatment and by at least 2.5% of the subjects (percentage given in parentheses) in each of the groups are listed. Similar terms from the different vocabularies were manually merged.			

**Table 2:** Clinical trial data downloaded from the Project Data Sphere portal.

**Trial**	**Study sponsor**	**No. of subjects** *	**Drug under investigation**	**Comparator arm treatment**	**ClinicalTrial.gov ID**
1	AstraZeneca	1627	Bicalutamide	Placebo†	NCT00657904
2	AstraZeneca	635	Zibotentan	Placebo†	NCT00626548
3	AstraZeneca	287	Bicalutamide	Placebo†	NCT00672282
4	AstraZeneca	657	Bicalutamide	Placebo†	NCT00673205
5	Centocor	47	Siltuximab (with mitoxantrone plus prednisone)	Mitoxantrone plus prednisone	NCT00385827
6	Celgene	226	Lenalidomide (with docetaxel plus prednisone)	Docetaxel plus prednisone	NCT00988208
7	Pfizer	278	Sunitinib (with prednisone)	Prednisone	NCT00676650
8	Sanofi	370	Cabazitaxel (with mitoxantrone plus prednisone)	Mitoxantrone plus prednisone	NCT00417079
All data files were downloaded on February 23rd, 2015.					

*Number of subjects for which survival data was available.

†In addition to background treatments including LHRH agonist, antiandrogen or orchiectomy.

## References

[d1] Project Data Sphere 2014 Prostat_AstraZe_1995_102

[d2] Project Data Sphere 2014 Prostat_AstraZe_2008_103

[d3] Project Data Sphere 2014 Prostat_AstraZe_1995_106

[d4] Project Data Sphere 2014 Prostat_AstraZe_1995_105

[d5] Project Data Sphere 2014 Prostat_Centoco_2006_98

[d6] Project Data Sphere 2014 Prostat_Celgene_2009_90

[d7] Project Data Sphere 2014 Prostat_Pfizer_2008_81

[d8] Project Data Sphere 2014 Prostat_Sanofi_2007_79

