# Supplementary material for: A patient-level data meta-analysis of standard-of-care treatments from eight prostate cancer clinical trials
Source: Sci Data. 2016 May 10;3:160027. doi: 10.1038/sdata.2016.27 (PMC4862324; doi:10.1038/sdata.2016.27)
Supplement: Supplementary Information [file sdata201627-s1.pdf]

Supplementary contents

Page number	Content
**2**	Supplementary figure 1
**3**	Supplementary figure 2
**4**	Supplementary figure 3
**5**	Supplementary Code

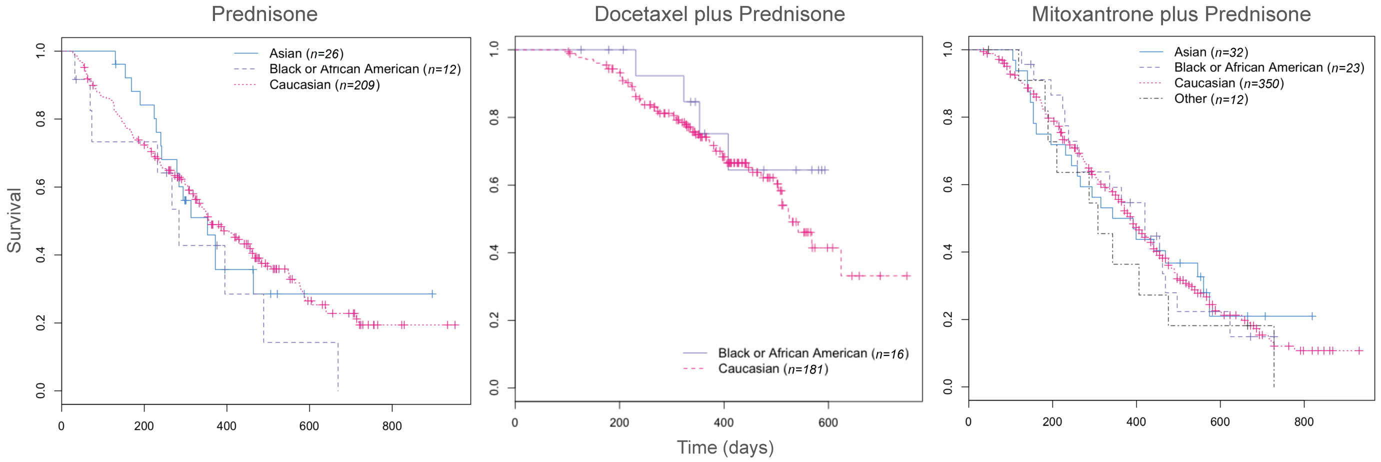

Supplementary figure 1: Survival of different racial groups within each chemotherapy treatment group (trials 5, 6, 7 and 8).

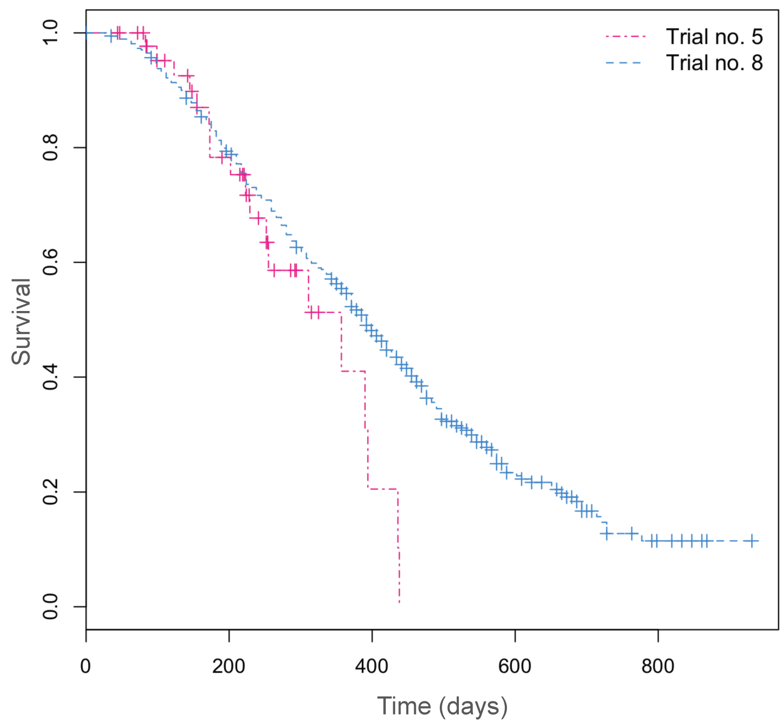

Supplementary figure 2: Survival of subjects from two different clinical trials (trials no. 5 and no. 8) but receiving the same treatment (Mitoxantrone plus prednisone).

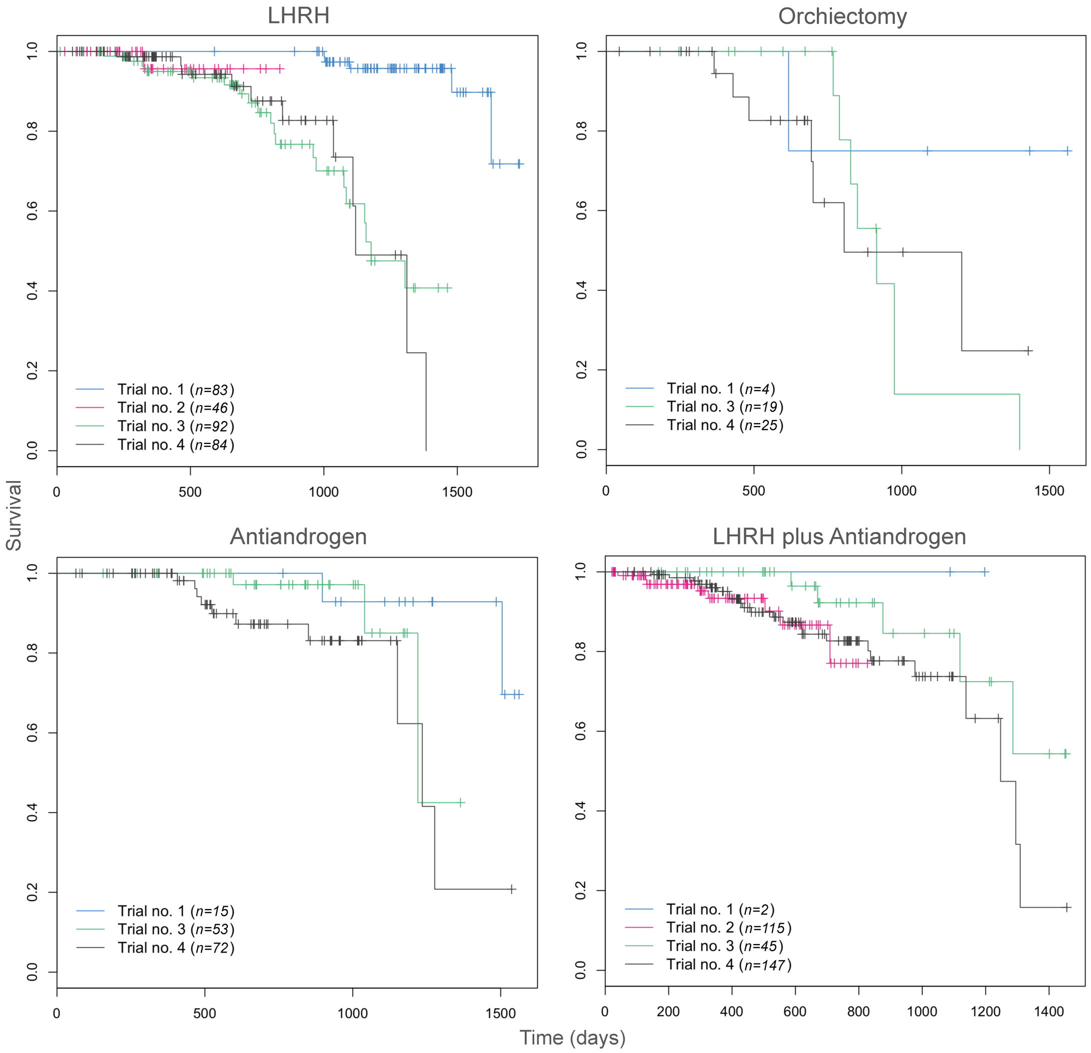

Supplementary figure 3: Survival of subjects from different clinical trials within each hormonal therapy treatment group (trials 1, 2, 3 and 4).

SUPPLEMENTARY CODE - .R ANALYSES SCRIPTS

```
library(survival); # load

in.data <- read.delim("data_file", header=TRUE, as.is=T);

####### Survival analysis ######

# 1
fit <- survfit(Surv(DAY, STATUS) ~ NEW_DRUG, data = in.data)
plot(fit, lty = c(4,2,3), col = colors()[c(117,128,176)], main="Prostate, stage IV",xlab="Days")
legend(320, 1.04, c("Docetaxel plus Prednisone (n=226)","Mitoxantrone plus Prednisone (n=419)","Prednisone (n=278)"), col = colors()[c(117,128,176)],lty = c(4,2,3), cex=1.1, bty="n")
survdiff(Surv(DAY, STATUS)~NEW_DRUG, data=in.data);
as.data.frame( summary(fit, times=c(365, 730, 1095, 1460, 1825) )[c("surv", "time", "strata")])
test <- coxph(Surv(DAY, STATUS)~NEW_DRUG + AGE + RACE + STUDY, data=in.data)
summary(test)

# 2
fit <- survfit(Surv(DAY, STATUS) ~ NEW_DRUG, data = in.data)
plot(fit, lty = 1:4, col = colors()[c(128,117,577,176)], main="Prostate, mixed stages",xlab="Days")
legend(50, 0.2, c("Antiandrogen (n=140)","LHRH (n=305)","LHRA plus Antiandrogen (n=309)","Orchiectomy (n=48)"), col = colors()[c(128,117,577,176)],lty = 1:4, cex=1.1, bty="n")
survdiff(Surv(DAY, STATUS)~NEW_DRUG, data=in.data);
test <- coxph(Surv(DAY, STATUS)~NEW_DRUG + AGE + RACE + STAGE + STUDY, data=in.data)
summary(test)
as.data.frame( summary(fit, times=c(365, 730, 1095, 1460, 1825) )[c("surv", "time", "strata")])


###### RACE COMPARISON #########

# prednisone
fit <- survfit(Surv(DAY, STATUS) ~ RACE, data = in.data)
plot(fit, lty = 1:4, col = colors()[c(128,595,117)], main="Prostate, stage IV",xlab="Days")
legend(390, 1.04, c("Asian (n=26)","Black or African American (n=12)","Caucasian (n=209)"), col = colors()[c(128,595,117)],lty = 1:4, cex=1.1, bty="n")
survdiff(Surv(DAY, STATUS)~RACE, data=in.data);

# docetaxel
fit <- survfit(Surv(DAY, STATUS) ~ RACE, data = in.data)
plot(fit, lty = 1:4, col = colors()[c(595,117)], main="Prostate, stage IV",xlab="Days")
legend(300, 0.15, c("Black or African American (n=16)","Caucasian (n=181)"), col = colors()[c(595,117)],lty = 1:4, cex=1.1, bty="n")
survdiff(Surv(DAY, STATUS)~RACE, data=in.data);

# mitoxantrone
fit <- survfit(Surv(DAY, STATUS) ~ RACE, data = in.data)
plot(fit, lty = 1:4, col = colors()[c(128,595,117,176)], main="Prostate, stage IV",xlab="Days")
legend(300, 1.04, c("Asian (n=32)","Black or African American (n=23)","Caucasian (n=350)","Other (n=12)"), col = colors()[c(128,595,117,176)],lty = 1:4, cex=1.1, bty="n")
survdiff(Surv(DAY, STATUS)~RACE, data=in.data);

# LHRH
fit <- survfit(Surv(DAY, STATUS) ~ RACE, data = in.data)
plot(fit, lty = 1:4, col = colors()[c(128,595,117)], main="Prostate, mixed stages",xlab="Days")
legend(390, 1.04, c("Afro-Caribbean (n=2)","Asian (n=1)","Caucasian (n=173)"), col = colors()[c(128,595,117)],lty = 1:4, cex=1.1, bty="n")
survdiff(Surv(DAY, STATUS)~RACE, data=in.data);
```

```
# Orchiectomy
fit <- survfit(Surv(DAY, STATUS) ~ RACE, data = in.data)
plot(fit, lty = 1:4, col = colors()[c(595,117)], main="Prostate, mixed stages",xlab="Days")
legend(300, 0.15, c("Caucasian (n=38)","Hispanic (n=1)", "Mixed (n=5)"), col = colors()[c(595,117)],lty = 1:4, cex=1.1, bty="n")
survdiff(Surv(DAY, STATUS)~RACE, data=in.data);

# Antiandrogen
fit <- survfit(Surv(DAY, STATUS) ~ RACE, data = in.data)
plot(fit, lty = 1:4, col = colors()[c(595,117)], main="Prostate, mixed stages",xlab="Days")
legend(190, 1.04, c("Afro-Caribbean (n=1)","Caucasian (n=118)","Hispanic (n=2)","Other (n=3)"), col = colors()[c(595,117)],lty = 1:4, cex=1.1, bty="n")
survdiff(Surv(DAY, STATUS)~RACE, data=in.data);

# Antiandrogen plus LHRH
fit <- survfit(Surv(DAY, STATUS) ~ RACE, data = in.data)
plot(fit, lty = 1:4, col = colors()[c(595,117)], main="Prostate, mixed stages",xlab="Days")
legend(190, 1.04, c("Caucasian (n=186)", "Hispanic (n=5)", "Other (n=1)"), col = colors()[c(595,117)],lty = 1:4, cex=1.1, bty="n")
survdiff(Surv(DAY, STATUS)~RACE, data=in.data);


## COMPARISON OF MERGED STUDIES WITHIN EACH TREATMENT GROUP ##

# LHRH
fit <- survfit(Surv(DAY, STATUS) ~ STUDY, data = in.data)
plot(fit, lty = 1:4, col = colors()[c(128,117)])
legend(930, 1.04, c("Trial no. 2 (n=92)","Trial no. 3 (n=84)"), col = colors()[c(128,117)],lty = 1:4, cex=1.1, bty="n")
survdiff(Surv(DAY, STATUS)~STUDY, data=in.data);

# Orchiectomy
fit <- survfit(Surv(DAY, STATUS) ~ STUDY, data = in.data)
plot(fit, lty = 1:4, col = colors()[c(128,117)])
legend(930, 1.04, c("Trial no. 2 (n=19)","Trial no. 3 (n=25)"), col = colors()[c(128,117)],lty = 1:4, cex=1.1, bty="n")
survdiff(Surv(DAY, STATUS)~STUDY, data=in.data);

# Antiandrogen
fit <- survfit(Surv(DAY, STATUS) ~ STUDY, data = in.data)
plot(fit, lty = 1:4, col = colors()[c(128,117)])
legend(1000, 0.15, c("Trial no. 2 (n=52)","Trial no. 3 (n=72)"), col = colors()[c(128,117)],lty = 1:4, cex=1.1, bty="n")
survdiff(Surv(DAY, STATUS)~STUDY, data=in.data);

# Antiandrogen plus LHRH
fit <- survfit(Surv(DAY, STATUS) ~ STUDY, data = in.data)
plot(fit, lty = 1:4, col = colors()[c(128,117)])
legend(920, 1.04, c("Trial no. 2 (n=45)","Trial no. 3 (n=147)"), col = colors()[c(128,117)],lty = 1:4, cex=1.1, bty="n")
survdiff(Surv(DAY, STATUS)~STUDY, data=in.data);
```
